# Optimization of RNA In Situ Hybridization for mRNA Localization Detection in Mature Tissue of Cucumber Seedlings

**DOI:** 10.3390/plants9111461

**Published:** 2020-10-29

**Authors:** Zixi Liu, Xi Hu, Jing Nie, Xiaojun Li, Qing Wang, Wenqian Liu, Tao Wang, Xiaohong Lu, Shunli Gao, Lihong Gao, Wenna Zhang

**Affiliations:** Beijing Key Laboratory of Growth and Developmental Regulation for Protected Vegetable Crops, China Agricultural University, Beijing 100193, China; 2016317010308@cau.edu.cn (Z.L.); huxii2013@163.com (X.H.); jnie@cau.edu.cn (J.N.); S20183172263@cau.edu.cn (X.L.); bolukesi@163.com (Q.W.); lwq08@cau.edu.cn (W.L.); B20183170825@cau.edu.cn (T.W.); luxh0317@gmail.com (X.L.); gshunli6689@163.com (S.G.); gaolh@cau.edu.cn (L.G.)

**Keywords:** cucumber, mature tissue of seedling, RNA in situ hybridization, mRNA localization

## Abstract

Cucumber (*Cucumis sativus* L.) is one of the main vegetable crops in China. The physiological cultivation mechanism and gene function characteristics of cucumber are of great significance to the construction of modern agriculture. Due to the low genetic transformation rate of cucumber, only in situ hybridization, which does not involve the progress of gene modified transformation, is convenient to study mRNA localization, so it is more suitable for determination on mRNA localization in the mature tissue of cucumber. At present, the existing in situ hybridization technology system is more suitable for cucumber meristem than for the mature tissue of cucumber seedlings. Therefore, we optimized the traditional plant in situ hybridization protocol. Taking a known gene CsNPF7.2 (Nitrate Transporter Families protein) as an example, we then optimized the steps of plant tissue culture, gene probe preparation, plant material sampling and fixation, preparation of cross section, hybridization pretreatment, hybridization incubation, chromogenic reaction, microscopy examination, and treatment after reaction termination in order to obtain a new RNA in situ hybridization technique suitable for identification on mRNA localization in mature tissues of cucumber seedlings. This optimized technique will ensure the yield of probes, the integrity of RNA molecules, and the clarity and integrity of plant tissue structure, which is conducive to the study of gene function and screening of key genes in cucumber.

## 1. Introduction

Cucumber (*Cucumis sativus* L.) is a main vegetable crop in China, which is the country with the largest cucumber production in the world. According to the statistics of the Food and Agriculture Organization of the United Nations (FAO), the proportion of cucumber annual yield in China accounted for 76.5% of the world in 2017, compared with 48.3% in 1961. Therefore, it is of great significance to study the physiological cultivated model and gene functional characteristics of cucumber for models of cultivation and management and the selection and breeding of high-quality new varieties. With the progress of genomics and bioinformatics, the whole-genome information of cucumber was finally deciphered [1]. However, most of these nucleotide sequences that were deciphered are new genes with missing annotation information. At this point, it is an urgent challenge for us to figure out the function of these new genes.

The function of genes is closely related to mRNA localization. Cucumbers are multi-cell type organisms, which are composed of different types of cells to form a variety of tissues and organs. Different tissues assume different physiological functions. Genes expressed in the same tissue are likely to function with similarities or close connections. For example, the main function of plant root hair is to increase root surface area, and its epidermal cells are mainly responsible for absorbing water and mineral nutrients from soil [2,3]. Vascular tissue of plants is responsible for transporting nutrients and metabolites required for plant growth and development from source to sink [4,5]. Therefore, the functional study of mRNA localization and screening of key genes are of guiding significance.

To date, mainstream research methods for plant tissue-level mRNA localization include in situ hybridization [6], histochemical localization [7,8], and fluorescent protein fusion labeling [9,10]. However, due to the influence of the regeneration system [11,12], cucumber varieties [13], and a high degree of genotype-dependent regeneration in cucurbits [14], the genetic transformation efficiency of cucumber is still low. Among the three methods, only in situ hybridization does not require construction for the cucumber transgene [6]. Therefore, it is more suitable for the study of mRNA localization identification at the cucumber tissue level. 

In situ hybridization technology originated in the middle of the last century, and the RNA in situ hybridization method used for mRNA localization of plant tissues is mainly based on previous studies [6,15]. The basic principle of this system is to use digoxin (DIG)-antisense probe recognizing sense mRNA for hybridization and then add alkaline phosphatase (AP), which can be coupled to DIG as a secondary antibody. The color substrates tetrazolium blue (NBT) and 5-bromo-4-chloro-3-indole-phosphate (BCIP) undergoes a chromogenic reaction and appears purple-red. Different plant materials have very different characteristics, and the application of in situ hybridization techniques between mature tissues and meristem of plant results in considerable differences. These experimental methods are particularly suitable for the study of plant meristems. In recent years, some in situ hybridization protocols have been optimized for plants [16,17], or in cucumber meristem [18] but none of these in situ hybridization systems can be directly applied to mRNA localization for root tissues of cucumber seedlings. Therefore, in the study to better identify mRNA localization, we optimized the RNA in situ hybridization technology for mature tissue of cucumber seedlings.

CsNPF7.2, a cucumber Nitrate Transporter Families protein, was reportedly involved in the loading, absorption, and utilization of nitrate in a previous study of our laboratory. The function of *CsNPF7.2* of which mRNA localized in the vascular bundle sheath and cambium was related to nitrogen stress. The abundance of *CsNPF7.2* mRNA was up-regulated under nitrogen deficiency condition, which promoted the growth of cucumber root xylem [19]. In this study, *CsNPF7.2* was used as an example to optimize the RNA in situ hybridization technology for mature tissue of cucumber seedlings.

## 2. Materials and Methods

### 2.1. Plant Materials

Tissue culture: “Xintaimici” cucumber seeds of uniform size, shape, and weight were screened and soaked in distilled water at 55~60 °C for about 30 min, and then the cucumber seed coat was peeled with small tweezers, being careful to avoid the tweezers touching the growth point of the seeds. Peeled seeds were sterilized with 75% alcohol for 30 s, washed 3 times with sterilized water, sterilized with 2.5% sodium hypochlorite for 10 min, and washed 3 times with sterilized water. The treated seeds were then placed on Murashige and Skoog (MS) solid medium and transferred to a growth chamber. The photoperiod was set as follows: The first stage was 28 °C dark culture for 24 h and the second stage photoperiod was 12 h, 28 °C during the day and 18 °C at night for 13 days.

### 2.2. RNA Probe Preparation

Based on the published consensus sequences in the Cucumber Genome Database (http://www.icugi.org/), PCR primers recognizing target gene was designed by using SnapGene 4.1.8 software as shown in Table 1. To obtain the transcription template, target gene was amplified by PCR in reactions containing PrimeSTAR Max DNA Polymerase (Takara, Beijing, China, Cat # R045A) and cDNA synthesized from cucumber leaves following the cycling conditions: 95 °C for 3 min, followed by 35 cycles of 95 °C for 30 s, 52 °C for 45 s, and 72 °C for 1 min. Then, according to the purchased T7/Sp6 in vitro transcription instructions guide, it was equipped with biotin digoxin labeled uracil nucleotide (Digoxigenin-11-UTP) in vitro transcription reaction system and left to react at 37 °C for 2 h. Here, we used published cucumber *CsNPF7.2* as an example [19].

Probe purification: First, 75 μL RNase-free H_2_O, 1 μL 100 mg/mL tRNA (Sigma, # 10109517001), and 1 μL DNase I (TaKaRa, Beijing, China) were added to the in vitro transcription product after the reaction was completed, and the DNA was incubated at 37 °C for 30 min to remove the DNA. Then, the RNA product was recovered using the anhydrous ethanol precipitation method by sequentially adding an equal volume of pre-cooled 4 M NH4Ac and 2 volumes of pre-cooled absolute ethanol, gently inverting to mix, and placing in a −20 °C refrigerator for 3–6 h. The product was centrifuged at 4 °C and 14,000 rpm for 10 min, and the supernatant was discarded. The precipitate was rinsed with 600 μL 70% ethanol and centrifuged at 4 °C 14,000 rpm for 7 min. The supernatant was discarded, the supernatant was drawn immediately, and the precipitate was left to dry. Then, 100 μL of RNase-free water was added to resuspend the precipitation, followed by 100 μL of 80 mM Na_2_CO_3_·120 mM NaHCO_3_ buffer and incubation at 60 °C for 50 min for hydrolysis. After the hydrolysis was completed, the RNA product was recovered again by alcohol precipitation. Finally, 40 μL of 50% formamide was added to dissolve the precipitate, and the mixture was placed in a refrigerator at −80 °C to store the in situ hybridization probe. Before the probe is placed in the ultra-low temperature refrigerator, Nanodrop or Qubit 3.0 can be used to measure the concentration of the RNA probe.

### 2.3. Reagents of the Pretreatment Process of In Situ Hybridization

The reagents required for this stage are all made up of RNase-free water. In addition to the commonly used reagents xylene, gradient ethanol (100–95–85–70–50%), 0.85% NaCl, ethylene diamine tetraacetic acid (EDTA), and phosphate-buffered saline (PBS) buffer, before the start of the experiment and according to the requirements of the experiment, proteinase K solution, 0.2% glycine solution, 4% formaldehyde solution, acetic anhydride solution, and other related solutions were prepared. Their formulas are as follows.

Protease K solution has an RNase-free water configuration. The working concentration of the three solution substrates was 100 mM Tris (pH 8.0), 50 mM EDTA, and 1 μg/mL proteinase K (Sigma, # p2308), and the dilution was calculated according to the relevant mother liquor purchased. The proportions were added sequentially, preheated at 37 °C one night in advance, and proteinase K was added as needed.

The 0.2% glycine solution was made up of RNase-free PBS buffer; 0.2 g glycine was added to 100 mL buffer. 

The 4% formaldehyde solution consisted of RNase-free water, high concentration PBS buffer, and 37% formaldehyde. After dilution, the working concentration of the solution substrate was 4% formaldehyde and 1× PBS.

Acetic anhydride solution was made up of RNase-free water. The working concentration of the solution substrate was 0.1 M triethanolamine, 0.5% NaCl, 48 mM HCl (made up of concentrated hydrochloric acid), and 0.025% acetic anhydride. Acetic anhydride was added before use, and the solution was stirred and mixed thoroughly.

### 2.4. Reagents of the First Hybridization Incubation (Primary Antibody)

The reagents required in this stage are made up of RNase-free water. Commonly used reagents formamide, tRNA, saline sodium citrate (SSC) buffer, and 50× Denhardt’s were directly purchased and used. Before the start of the experiment, 10× salts, 50% dextran sulfate, RNA hybridization solution, 2× SSC/50% formamide, and other related solutions were prepared. Their formulas are as follows:

The 10× salts solution was made up of RNase-free water. The working concentration of the solution substrate was 49 mM Na_2_HPO_4_ 51 mM NaH_2_PO_4_ buffer (pH 6.8), 3 M NaCl, 50 mM EDTA, and Tris·HCl (pH 8.0), which were stored at −20 °C for a long time.

The 50% dextran sulfate was mixed in RNase-free water heated at 60 °C. Then, 0.5 g of dextran sulfate was mixed and dissolved in 10 mL RNase-free water and stored at −20 °C for a long time.

RNA hybridization solution was made up of RNase-free water. The amount of hybridization solution per slice was 120 μL, and the amount of substrate added to 1 mL of RNA hybridization solution was 500 μL formamide (100%), 200 μL 50% dextran sulfate, 170 μL RNase-free water, 100 μL 10× salts, 20 μL 50× Denhardt’s, and 10 μL 100mg/mL tRNA. The corresponding dosage was configured according to the number of hybridization slices. Dextran sulfate is viscous, so it was thoroughly mixed by pipetting, and the pipetting gun suction speed was carefully controlled.

The 2× SSC/50% formamide was made up of RNase-free water, 20× SSC buffer and 100% formamide. After dilution, the working concentration of the solution substrate was 2× SSC and 50% formamide.

### 2.5. Reagents of Second Hybridization Incubation (Secondary Antibody)

In addition to the commonly used reagents, this stage of the experiment required the purchase of 10× tris-buffered saline (TBS) buffer, RNase A, bovine serum albumin (BSA), skimmed milk powder (Roche, Shanghai, China), Triton X-100 (Sigma, Shanghai, China), DIG-AP (Roche, Shanghai, China), and Chromogenic substrate NBT/BCIP (Roche, Shanghai, China). Before starting the experiment, five buffers, Buffer 1 (0.2× SSC), Buffer 2, Buffer 3, Buffer 4, Buffer 5, and two reaction solutions, AP antibody solution and NBT/BCIP solution showing substrate, were created. The solutions at this stage can be prepared by ordinary distilled water, and their formulas are as follows:

Buffer 1 solution consisted of 20× SSC diluted with distilled water to 0.2× SSC according to the experimental dosage.

Buffer 2 solution was made of distilled water. The working concentration of the three solutions was 0.5 mM NaCl, 10 mM Tris·HCl (pH 7.5), and 1 mM EDTA. It was made up according to the experimental dosage.

Buffer 3 solution was made of 1× TBS buffer solution at 60 °C. The 100 g 1× TBS buffer solution was mixed with 1 g of skimmed milk powder (Roche, Shanghai, China# 11096176001) and heated continuously at 60 °C on a magnetic stirrer to ensure that the skimmed milk powder was fully dissolved.

Buffer 4 solution was made of TBS buffer and distilled water. The working concentration of the solution substrate was 1× TBS, 1% BSA (Sigma, Shanghai, China# sre0096), and 0.3% Triton X-100 (Sigma, Shanghai, China# t8787), and the appropriate dosage is made up according to the requirements of the experiment. The solution was stirred at room temperature on a magnetic stirrer to mix thoroughly.

Buffer 5 solution was made of distilled water. The working concentration of the solution substrate was 0.1 M Tris·HCl (pH 9.5), 100 mM NaCl, and 50 mM MgCl_2_, and the appropriate dosage was made up according to the requirements of the experiment and well mixed.

To create the AP antibody solution, DIG-AP antibody (Roche, Shanghai, China#11093274901) was diluted 1250 times with Buffer 4; that is, 10 mL Buffer 4 solution was added to 8 μL DIG-AP antibody. The required configuration was calculated based on the amount of 200 μL AP antibody solution per slice.

The NBT/BCIP display substrate solution was made of Buffer 5 solution. The working concentration of the two chromogenic substrates was 225 μg/mL NBT (Roche, Shanghai, China#11383213001) and 175 μg/mL BCIP (Roche, Shanghai, China#11383221001). The dosage was configured according to the experiment requirements.

## 3. Results and Discussion

### 3.1. The Existing In Situ Hybridization Technology Is Not Suitable for mRNA Localization Detection in Cucumber Mature Tissues 

The function of genes is very important for cucumber research. On the one hand, the published whole-genome information provides great convenience for the research of the molecular mechanism of cucumber. On the other hand, due to the low genetic conversion rate of cucumber, the rapid screening of key genes from many candidate genes has become a new challenge.

**Figure 1:**
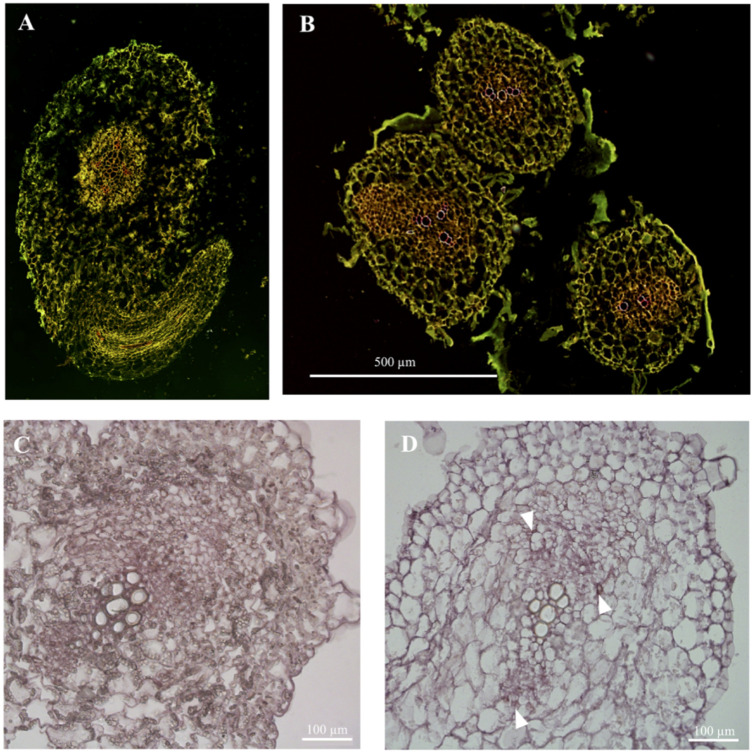
Comparison between the existing in situ hybridization technology system and the technical system of the method. (**A**) The existing in situ hybridization technology system can fix the root tissue shrinkage of cucumber seedlings, and the results are incomplete; (**B**) The in situ hybridization protocol of present method is applied to the cucumber seedling root system, with complete tissue structure and clear cell outline; (**C**) The cell content of the existing in situ hybridization protocol interferes with the hybridization signal; and (**D**) The in situ hybridization protocol of the present method is applied to the mature tissue of cucumber seedling. The hybridized signal was clearly visible (the white triangle points to the hybridization signal).

In situ hybridization is more suitable for mRNA localization at the tissue level in cucumber. Currently, the existing in situ hybridization technology system applied to plants is suitable for meristems of plants (for example, development of leaves and shoots, differentiation of pistils and stamens, development of embryo or ovule) as well as most mature tissues of plants (for example, apple seedling roots). These plant materials are relatively thick, and they either harden after encountering the fixing solution or they have a higher degree of lignification. The roots and stems of cucumber seedlings are too young, and the hybrid molecular signal of mature tissue is different from the meristematic tissue, so the following problems occur when directly applying the existing system:

(1) Plant material cultivation: The roots of cucumber seedlings cultivated with traditional peat, vermiculite, and perlite formulations adhere to many substrate impurities that are difficult to remove. Violently rinsing can completely remove impurities but also destroy the tissue structure of cucumber roots.
(2) Gene probe preparation: The traditional system uses the target gene cDNA fragments to ligase into pGEM-T or pGEM-T Easy vector and then obtains the in vitro transcription template by linearized vector digestion and purification. This method not only increases the economic cost, but the yield of the template is directly affected by the ligation efficiency and digestion efficiency.
(3) Sampling and fixation for plant material: The stems and roots of cucumber seedlings are very young, with low density, and are difficult to precipitate by vacuuming. The traditional cutting pattern, fixative formulation, gradient ethanol dehydration system, and paraffin embedding method are not suitable, resulting in tissue shrinkage, damage, and incomplete structure (Figure 1).
(4) Preparation of hybridization sections: The traditional system is optimized for this step, and this step has had an important influence on the recent in situ hybridization results of mature tissues of cucumber seedlings.
(5) Hybridization pretreatment: The traditional system does not optimize the samples processed in this step, and multiple reagent solutions are used multiple times at this stage, resulting in a large consumption of resources.
(6) Hybrid incubation: The traditional system is not optimized for this step, and the technical method of this step has a direct impact on the accuracy of the mRNA localization results of mature tissues of cucumber seedlings.
(7) Chromogenic reaction microscopy: The traditional system is not optimized for this step, and this step has a direct effect on the specificity of the mRNA localization in the mature tissue of cucumber seedlings
(8) Post-reaction processing: The traditional system requires TE buffer to stop the chromogenic reaction, before rinsing with xylene and ethanol according to the gradient, or a picture must be taken after stopping the reaction with clean water. However, the signal of hybrid molecules in mature tissues of cucumber seedlings is distributed in a “point”, which is different from the signal of hybrid molecules distributed in meristems in a “face” distribution. Therefore, the hybridization signal is disturbed by a large number of cell contents and cannot be clearly seen, and it is also easily decolorized by rinsing with ethanol or xylene (Figure 1).

These limitations require a new set of in situ hybridization optimization schemes suitable for mature tissues of cucumber seedlings that solve the inadequate technology in the steps of cucumber seedling material sourcing, sampling, fixation, probe preparation, hybridization process, and post-processing of hybridization color development when applying the existing system.

The stability of RNA and the integrity of tissue sections are the necessary factors for the success of all RNA in situ hybridization experiments. The yield of probes, the sensitivity of detection, the pretreatment of hybridization, incubation, microscopic examination, and the treatment after reaction termination also has a great influence on the hybridization results. Therefore, we optimized RNA in situ hybridization for mature tissue of cucumber seedlings. The following is optimized in situ hybridization technology and a comparison with the previous method.

### 3.2. Optimization of Cultivation Method and Probe Construction Method

In traditional technology the formula of peat, vermiculite, and perlite were used for substrate cultivation. Plant tissue easily adheres to matrix impurities, and the plant tissue structure is easily damaged upon removal. Using MS solid medium can effectively reduce impurity attachment, and it is easy to clean, preventing damage to the plant tissue structure.

**Figure 2:**
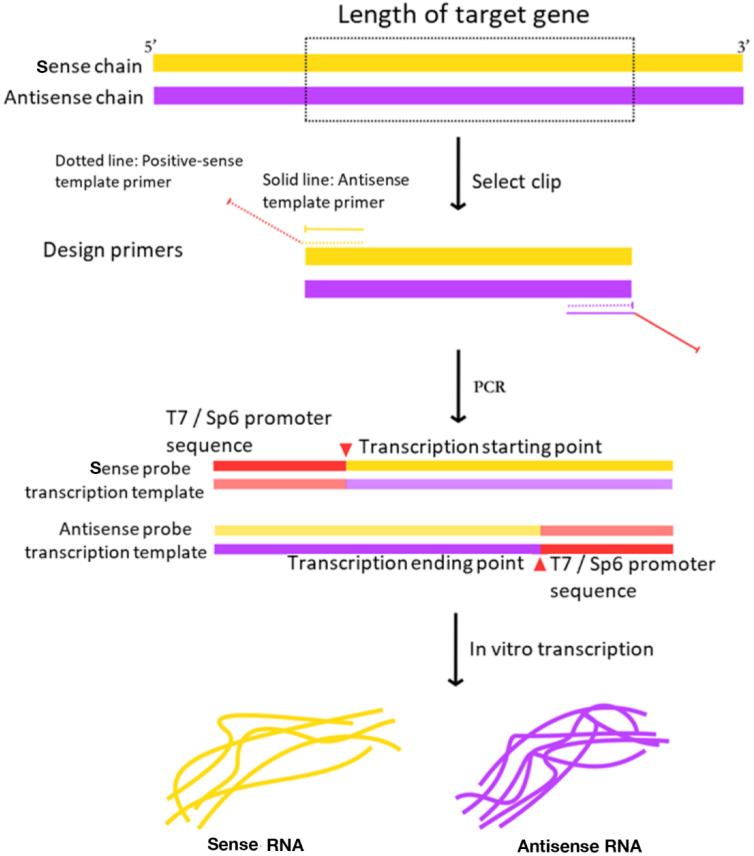
Schematic of the optimized RNA probe with target gene preparation and generation. The transcriptional probe is consisting of two parts: RT-PCR fragment and T7/SP6 promoter sequence. The sense probe and the antisense probe are ligated in opposite directions to the promoter. The sense probe is the negative control of the target gene, and the antisense probe is the complementary sequence of the target gene. According to the mRNA sequence length of the target gene, the sequence of the target gene initiated from GG and with a GC content of 40–70% was selected as a template fragment. The length of template fragment is better in the rage of 500–1500 bp. The dotted line indicated primers for sense probe and the solid line indicated primers for antisense probe.

Meanwhile, in the traditional system, by purifying the digested linear vector product, RT-PCR fragments with target gene was ligated in the vector of pGEM-T or pGEM-T Easy to obtain the in vitro transcription template. This method not only increases the economic cost, but the yield of the template is also directly affected by the ligation efficiency and digestion efficiency. However, in the optimized protocol RT-PCR amplified the sense probe and antisense probe transcription template of target gene by high fidelity PCR and then transcribed in vitro to obtain the RNA probe (Figure 2). And it can avoid the steps of recovering the enzyme products, avoid the linking and enzyme cutting, and reduce the influence of the efficiency of the connection and enzyme digestion.

### 3.3. Optimization of Sample Fixation of Cucumber Seedling Tissues

**Figure 3:**
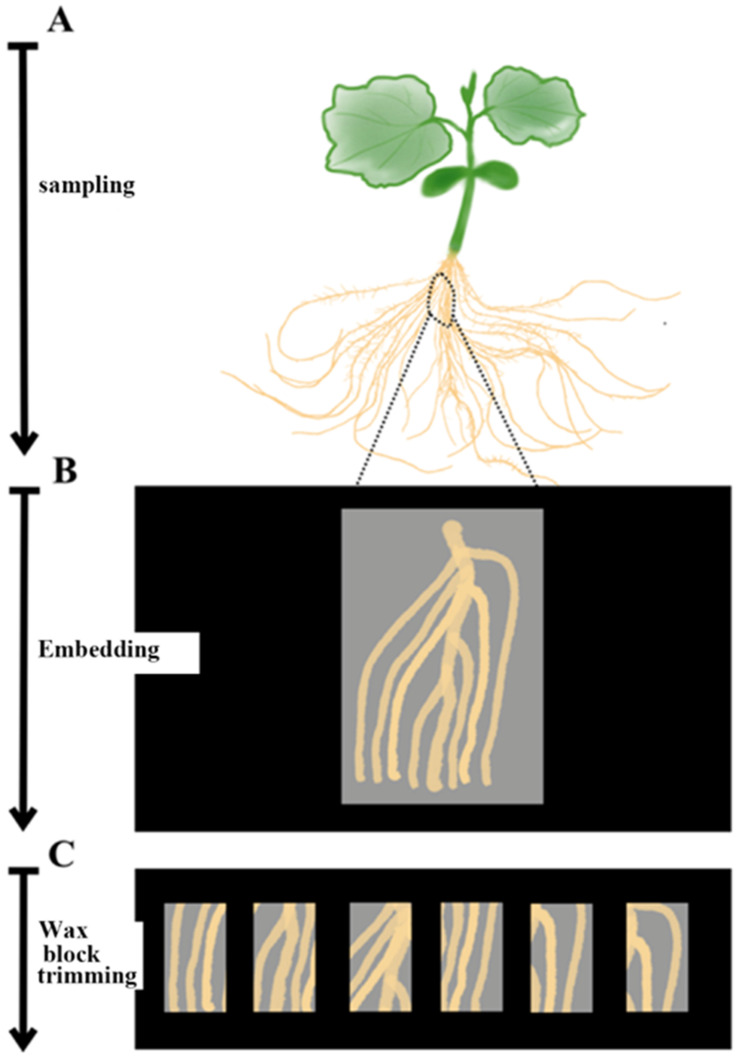
Schematic of the optimized sampling method of cucumber seedling root tissue. Due to the less lignification in stems, the small and softened part of roots of cucumber seedlings (**A**) and the stem with a length of 3–4 cm and the root segments containing lateral roots (**B**) were sampled and then cut into smaller pieces (**C**) before section.

Cucumber seedlings were sampled on ice. Due to the less lignification in stems of cucumber seedlings, the small and softened part of roots, the stem with a length of 3–4 cm and the root segments containing lateral roots were cut into smaller pieces for tissue sampling before section (Figure 3).

The removed plant material was quickly put into a pre-cooled formaldehyde-acetic acid-ethanol (FAA) fixing solution (improved formula: 30 mL absolute ethanol, 10 mL 37% formaldehyde, 5 mL glacial acetic acid, 55 mL RNase-free water) and pumped into a vacuum with the vacuum pump for 18–20 min. The pump was allowed to retake air to return to atmospheric pressure, and the solution was replaced by a new FAA fixing solution, which was shaken at 4 °C in a horizontal shaker overnight. The sample was stored at 4 °C for 1 week. The dehydrated samples were shaken on a horizontal shaker at 4 °C. The ethanol concentration gradients used were 50%, 70%, 85%, 95%, 100%, 100%, and 100% (made from RNase-free water). From low concentration to high concentration, a small amount of eosin stain (used to distinguish paraffin and sample during sectioning) was added to each gradient until the plant sample was dyed pink. The dehydration time of each gradient was improved to about 35 min, and the last 100% ethanol gradient was stored overnight.

Xylene was also used instead of ethanol to shake in a room temperature horizontal shaker. Concentrations of 100% ethanol, 50% ethanol/50% xylene, 100% xylene, 100% xylene, 100% xylene, and 100% xylene were used in this order. The replacement time of each solution was about 30 min, and the last 100% xylene solution was stored overnight. Xylene was replaced with paraffin in the 60 °C incubator. The xylene was directly replaced with paraffin melted in advance, and this step was repeated for 4 days, once a day.

The sample was embedded on an ultra-clean bench treated with RNase scavenger. An ordinary enzyme-free square plastic petri dish as an embedding box was placed on ice. A clean paraffin melt of about 1 cm in thickness was added, and the sample was quickly placed in it. A flame was used to burn the metal tweezers to drive away from the bubbles around samples. The paraffin contacted fully with the sample. After the paraffin had solidified, it was stored in a −20 °C refrigerator.

In the improved technology, we cut the plant tissue into larger pieces and cut the embedded sample into smaller tissue samples before slicing, so as to avoid the damage to cucumber tissue. Compared with the mature tissue, we reduced the ethanol content in FAA fixed solution, in order to prevent material shrinkage and reduce the damage to plant tissue. In the process of dehydration, we set three 100% ethanol concentration gradients and slightly extended the dehydration time to facilitate full dehydration. A small amount of eosin dye was added into the ethanol solution to make the plant tissue pink, which was convenient for the separation of paraffin and sample. The whole embedding process was carried out on an enzyme-free vertical clean bench, and an enzyme-free plastic box was used to avoid enzyme damage to the RNA structure and plant tissue structure.

### 3.4. Optimization of Embedding and Paraffin for Cucumber Seedlings In Situ Hybridization

The hybridization signal of mature tissue is different from that of the meristem. Signal molecules are scattered in a single cell. The tissue sections of the sample should not be too thin, otherwise it will reduce the number of target molecules fixed on the tissue. Mature tissue sections of cucumber seedlings with a thickness of 10–12 μL are suitable for in situ hybridization experiments.

**Figure 4:**
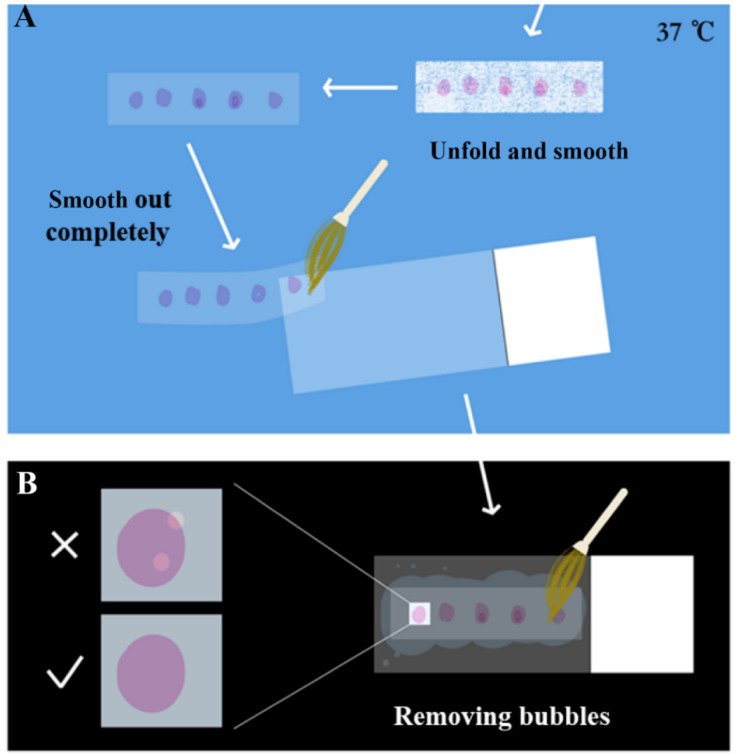
Schematic tips of the optimized process of unfolding and smoothing sections. (**A**) The sections softened and smoothed out with paraffin completely, until the color changed from white to almost transparent. Then, the section was smoothed out again gently with a brush on the slide. (**B**) Pressed lightly with the brush to drive off the air bubbles, and then absorbent paper was used to gently absorb the water to reduce the drying time and improve the effect of drying, avoiding the separation of the sample from the slide later.

The paraffin section samples produced by the microtome were screened before striping. A blade was used to divide the paraffin section into a length suitable for the placement of the slide. The section was gently laid flat on the slide and placed under the microscope for simple microscopic examination to screen the required tissue sections. Sections with incomplete structures such as tissue fragmentation, voids, and blade scratches were discarded. After screening, the sections were gently placed in 37 °C RNase-free water for spreading. As shown in Figure 4, the sections were softened and smooth out with paraffin completely, until the color changed from white to almost transparent. Then, the section was smoothed out again gently with a brush on the slide, pressed lightly with the brush to drive off the air bubbles, and then absorbent paper was used to gently absorb the water to reduce the drying time and improve the effect of drying, avoiding the separation of the sample from the slide later.

During the slicing process, wax stains on the blade were checked and cleaned to avoid fragmentation caused by scratches.

Block Trimming: Switch on the alcohol burner and placing a clean newspaper pad on the table, using the 2-mL centrifuge tube rack and 2-mL centrifuge tube, the successfully embedded sample was removed, and a surgical blade was used to cut the sample into a 0.5 × 0.5 × 0.5 mm wax block containing the sample (excess paraffin was removed; the closer to the edge of the sample, the better). A small piece of crushed paraffin was placed on the centrifuge tube cover, the paraffin was melted with a blade heated with an alcohol flame, and then the freshly cut wax block was quickly placed on the paraffin. As the paraffin solidified, the wax block was fixed on the centrifuge tube. Sample names were marked on the centrifuge tube.

Section: After switching on the microtome, the sample head was returned to the original position, setting the section thickness to 10 µm, then the successfully repaired sample was placed in the microtome, adjusting the position of the blade to form a cut surface with the sample. The microtome was then rotated, using a brush to help push away the paraffin section that forms the strip to prevent the samples from sticking together.

Section Unfolding and Paving: A fine brush was used to place the sample on clean A4 paper, and the sample was cut into small pieces with a blade and then placed on a glass slide. A microscope was used for preliminarily screening to verify that the tissue was complete, without damage, and of the desired tissue structure. Then, the selected fragments were carefully placed into a 37 °C water bath spreader, and the sample was carefully flattened. Finally, the sample was lifted with the slide glass to make it stick to the slide glass, and the excess water around the sample with was carefully absorbed absorbent paper. The sample was pushed lightly with a brush to drive away from the bubbles.

Slides Drying: Slides that were successfully spread were placed in a 42 °C oven overnight, so that the samples were completely pasted onto the slides to ensure that the next step of dewaxing did not cause the tissue to fall off.

The hybridization signals of mature tissue are different from those of the meristem, which are scattered in a single cell. The tissue section of the sample should not be too thin, otherwise the number of target molecules fixed on the tissue will be reduced. Therefore, the thickness of 10 µm was selected to increase the number of target molecules in the tissue as much as possible.

### 3.5. Optimization of the Pretreatment Process of In Situ Hybridization

Hybridization pretreatment deparaffinizes and decolorizes the paraffin-coated tissue samples and treats the protein that wraps the target molecule, so that the target molecule is fully exposed. Therefore, all utensils should be treated with RNase scavenger, and all reagents should be made up of RNase-free water.

**Figure 5:**
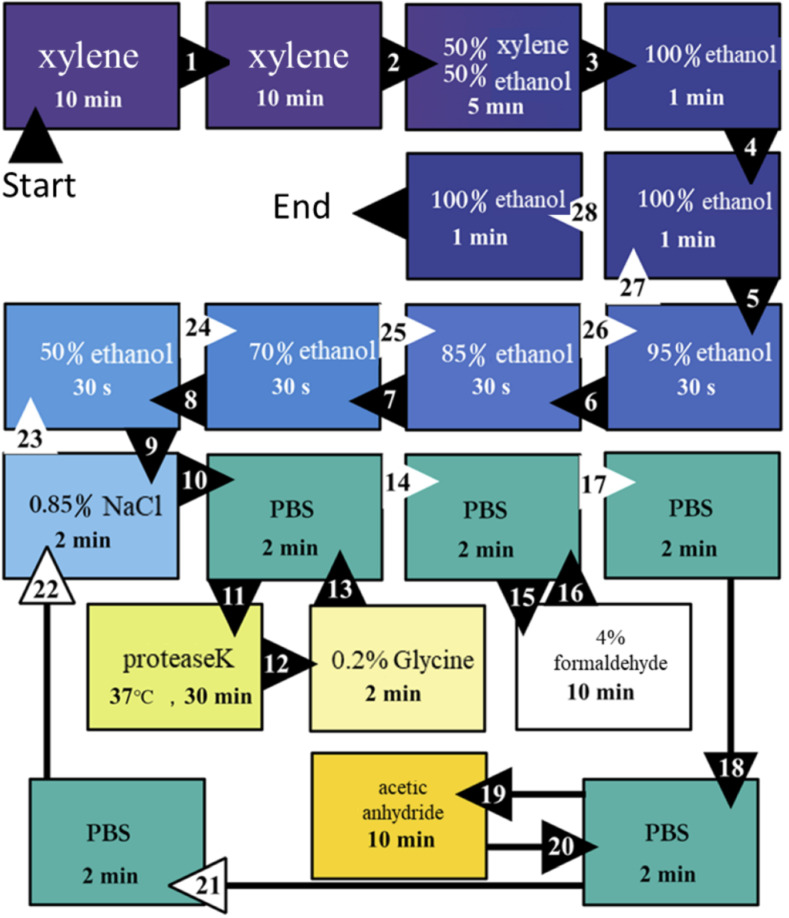
Schematic process of the optimized hybridization pretreatment.

This process repeatedly used PBS buffer, gradient ethanol, etc. The process was optimized as shown in Figure 5. The final sample was stored in a 4 °C refrigerator and placed in a staining tank sealed with parafilm, with a small amount of absolute ethanol at the bottom.

In the pretreatment stage, the concentration gradient of alcohol was added to wash away the excess xylene plant tissue, which could be fully rehydrated. In addition, 0.85% NaCl was directly used to reduce the consumption of reagents.

### 3.6. Optimization of the Process of In Situ Hybridization

#### 3.6.1. The First Hybridization Incubation (Primary Antibody)

Diluted Probe: The amount of 30-μL diluted probe per slice (2-μL probe + 28 μL 50% formamide solution) was calculated, and the sense and antisense probes were diluted.

**Figure 6:**
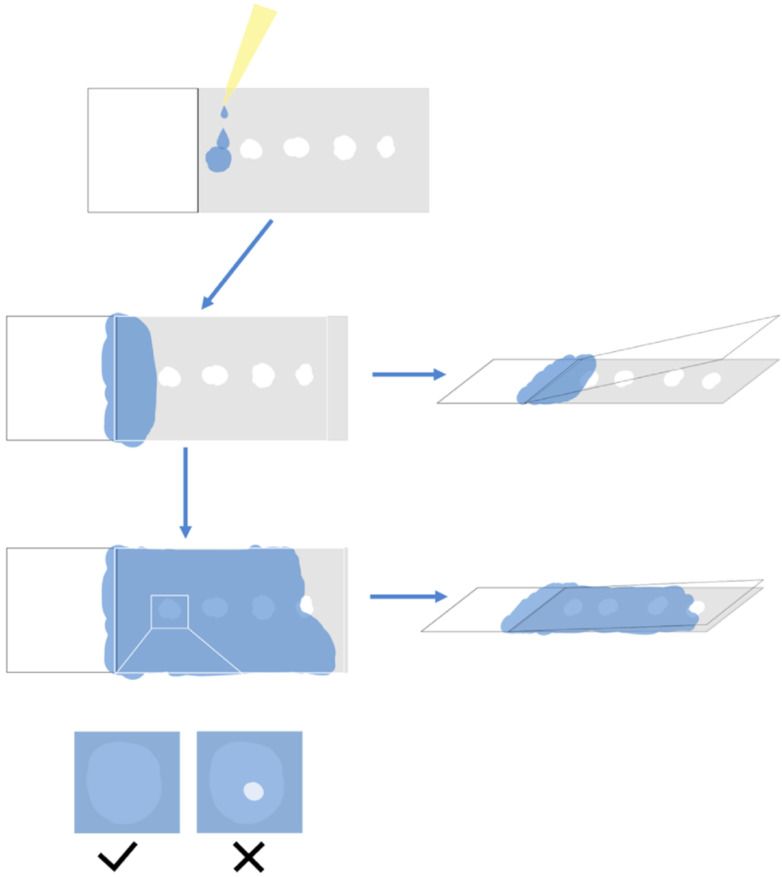
Schematic of the key technical points in the sealing of optimized hybridization incubation. The RNA hybridization liquid drops on the position shown with blue color, and then gently pressing down one side of the cover glass on this side and press it with the left hand to make the cover glass fit the RNA hybridization liquid. Hold the cover glass with the right hand and slowly put down the other layer. The RNA hybridization liquid is pushed away slowly with the cover glass. The moving speed of the cover glass must be slower than that of the RNA hybridization liquid, especially the viscous anti RNA hybridization liquid with glucosamine sulfate.

First, the slide was moved from the staining rack to the slide plate and dried at room temperature for 5–10 min until the tissue sample was pure white. At the same time, the diluted probe was denatured at 80 °C for 2 min, immediately placed on ice for 2–3 min, and separated for 30 s. Then according to the calculation of 120 μL/piece, the corresponding RNA hybridization solution was added and mixed with a pipette tip. Next, an appropriate amount of 2× SSC/50% formamide solution was added to the immunohistochemically wet box. Then, as previously described, 135 μL of hybridization mixture solution was added to each slide. After the solution completely covered the sample, it was covered with a slip and then put into the wet box. Finally, after all the slides are placed in the wet box, the wet box was sealed with tape and placed in an incubator at 50–55 °C overnight (Figure 6). In the optimized technique, the probe was deformed at 80 °C for 2 min, then cooled rapidly and left for 30 s to prevent its refolding. 

#### 3.6.2. Second Hybridization Incubation (Secondary Antibody)

**Figure 7:**
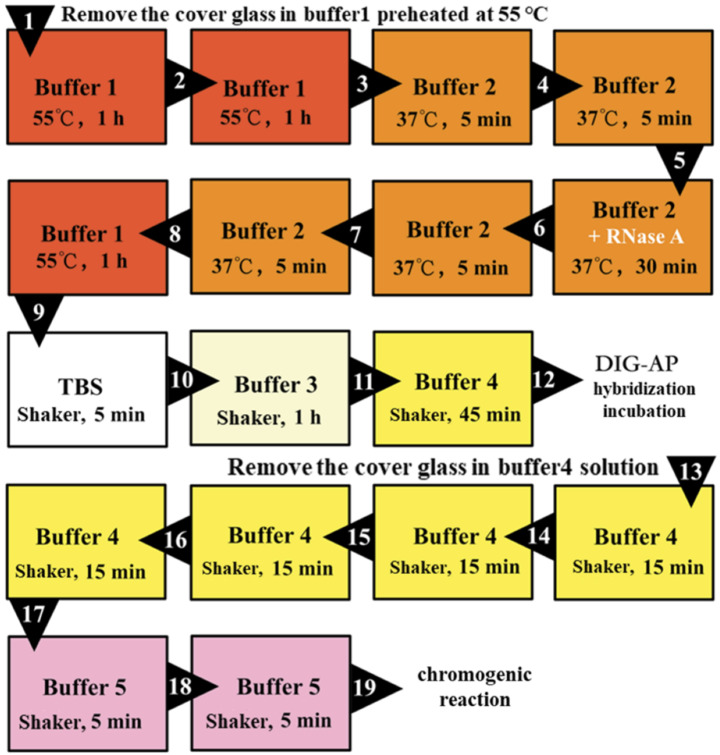
Post-reaction processing diagram of the optimized in situ hybridization.

As shown in Figure 7, the samples were processed before the second hybridization incubation, and then each slide was washed with 100 μL of the AP antibody-containing solution as previously described. Then, 100 μL of AP antibody solution was used for mounting and placed in a wet box with Buffer 4 to incubate at room temperature for 2 h. After the second hybridization incubation, the coverslips were washed off, then washed four times with Buffer 4 for 15 min each time and two times with Buffer 5 for 5 min each time, as shown in Figure 7.

Finally, in a dark environment, the sample section was rinsed once with 120 μL NBT/BCIP chromogenic substrate solution, and the slides were pasted together in pairs, transferred to a Coplin cup with 2 mL of NBT/BCIP chromogenic substrate solution at the bottom, wrapped and sealed with foil, and placed in a dark environment. The chromogenic reaction was performed at room temperature.

The processing time of Buffer 3 was extended to improve the ability of specific recognition.

#### 3.6.3. Chromogenic Reaction and Microscopy Examination

The in situ hybridization system of the mature tissue of cucumber seedlings is not suitable for terminating the chromogenic reaction directly after three days. In order to minimize background noise and increase the specificity of target mRNA localization, hybridization signal molecules should be discovered as soon as possible, and the earlier the signal appears as noise, the less likely it is to be discovered.

We need to adjust the chromogenic reaction and microscopy examination time according to RNA abundance. For high relative abundance transcripts (the Cq value is 15 ≤ Cq < 25, the Cq value of internal reference gene normally is around 15), microscopic examination should be conducted within 6 h after chromogenic reaction. For general relative abundance transcripts (the Cq value of target gene is 25 ≤ Cq ≤ 30), the microscopic examination was performed twice a day in the morning, afternoon or evening on the next day. The lower the Cq value and the earlier the microscopic examination that was required to be carried out. For example, the qPCR Cq value of *CsNPF7.2* was 30.49, and the Cq value of the internal reference gene actin was 14.38, it was a low abundant transcript, but it still can be detected.

Light was avoided during microscopy examination. The sticky slides were separated in the chromogenic substrate buffer (Buffer 5), making temporary slides with the buffer, which were then placed under the microscope to check the hybridization signal. If a clear hybridization signal (purplish red) molecule was observed, the chromogenic reaction could be terminated. Otherwise, the sample was rinsed again with the chromogenic reaction solution. Then, the sticky slide was put back into the Coplin cup to continue the reaction.

In the chromogenic reaction, the slides were adhered together to prevent the slices from falling off.

#### 3.6.4. Post-Reaction Processing

The cells of the mature tissues of cucumber seedlings are rich in inclusion impurities. They are almost insoluble in water, ethanol, and xylene, and black-like impurities under the microscope seriously interfere with the hybridization signal (Figure 1D).

For the tissue samples where the hybridization signal was observed, the chromogenic reaction was terminated with clean water and then left to dry at room temperature. Finally, each slide was directly mounted with a mixture of 90 μL neutral resin/xylene (1:1). The samples after mounting were hardly affected by the inclusion impurities, the hybridization signal was clear, and the experimental results were permanently preserved.

### 3.7. Detection Result

**Figure 8:**
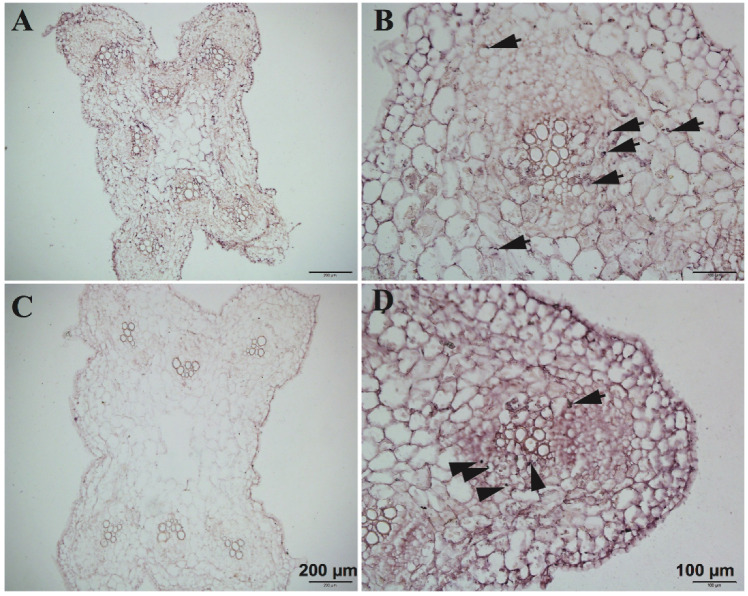
RNA in situ hybridization of cucumber *CsNPF7.2* in cucumber root and hypocotyl. Cross-section of hypocotyl (**A**,**B**), root (**D**) and sense probe controls of hypocotyls (**C**) of 15-day-old cucumber seedling. Red scales bars in all images are 100 μm and 200 μm. RNA signals are indicated by black arrowhead.

In the experiment, we used *CsNPF7.2* to study the optimization of RNA in situ hybridization. The function of *CsNPF7.2* is related to nitrogen stress. Under nitrogen deficiency, the expression of *CsNPF7.2* is up-regulated, which could promote the growth of xylem in cucumber root [19]. We observed the results of in situ hybridization (Figure 8) of *CsNPF7.2*, which was highly expressed in vascular cambium and parenchymal cells around the xylem of cucumber root.

The optimized RNA in situ hybridization technology can be applied to mRNA localization research of mature tissue of plant seedlings, or rhizome tissue with higher lignification degree and thicker diameter. This method can also be used when the content of the cell is large or the hybridization molecules are distributed in the form of dots.

A comparison has completely shown the detailed steps difference between traditional methods and improved methods and the advantages of improved methods (Table 2).

## 4. Conclusions

We reported an optimization of RNA in situ hybridization for mRNA localization in mature tissues of cucumber seedlings to avoid the effects of lignification, coarse diameter, dot-like signal distribution, and more cell contents. Specifically, we optimized each step of RNA in situ hybridization technology to avoid damage to the plant tissue structure, maintain the integrity of RNA, and improve the yield of the probe, so as to obtain a clearer and complete hybridization signals. It concluded that the efficiency of RNA in situ hybridization depends largely on the yield of the probe, the stability of RNA, and the integrity of plant tissue structure in many experimental steps. RNA in situ hybridization of *CsNPF7.2* was carried out to evaluate the optimized protocol. The results of hybridization showing a clear and complete cell structure proved the availability of protocol.

## Figures and Tables

**Table 1:** *CsNPF7.2* (Nitrate Transporter Families protein) gene in situ hybridization probe primer table.

*CsNPF7.2*	Forward Primers	Reverse Primers
Positive-sense probe	TAATACGACTCACTATAGGGGGCGATGAACAAA	CCAACAAATGGATCGAGAACGCGA
Antisense probe	GGCGATGAACAAACGCCCTG	ATTTAGGTGACACTATAGAACCAACAAATGGATCGA

Note: The positive-sense probe is connected to the T7 promoter and the antisense probe is connected to the Sp6 promoter. The underlined sequences in the table are the promoter sequence.

**Table 2:** Comparison of traditional and improved in situ hybridization protocol.

Steps	Traditional Protocol		Optimized Protocol	
	Protocol	Disadvantage	Protocol	Advantage
Plant material cultivation	The substrate cultivation: peat, vermiculite and perlite (2:1:1).	Many substrate impurities are difficult to remove. The plant tissue structure is easily damaged when removing the matrix impurities.	Murashige and Skoog (MS) solid medium	Effectively reduce impurity attachment. It is easy to clean so that prevent damage to the plant tissue structure.
Gene probe preparation	By purifying the digested linear vector product, RT-PCR fragments with target gene was ligated in the vector of pGEM-T or pGEM-T Easy to obtain the in vitro transcription template.	Not only increases the economic cost, but the yield of the template is also directly affected by the ligation efficiency and digestion efficiency.	RT-PCR amplified the sense probe and antisense probe transcription template of target gene by high fidelity PCR and then transcribed in vitro to obtain the RNA probe.	Avoid the re-ligation of digest products and reduce the influence of the efficiency of ligation and digestion.
Sampling and fixation for plant material	The plant tissue was cut into smaller pieces with a length of 3–4 mm. FAA fixing solution (90 mL 50%/70% ethanol, 5 mL glacial acetic acid, 5 mL 37% formaldehyde). There are fewer dehydration gradients and each gradient is 30 min. Ethanol used for dehydration was without eosin dye. No emphasis on enzyme-free throughout the embedding.	It‘s difficult to precipitate by vacuuming. The traditional method is not suitable for the stems and roots of cucumber, resulting in tissue shrinkage, damage, and incomplete structure.	The stem and the root segments containing lateral roots were cut into smaller pieces with a length of 3–4 cm. FAA fixing solution (30 mL absolute ethanol, 10 mL 37% formaldehyde, 5 mL glacial acetic acid, 55 mL RNase-free water). Extend each dehydration gradient time to 35 min, and the last gradient time overnight. Embedding was carried out on an enzyme-free clean bench.	Cut the tissue into larger pieces before slicing and then cut the embedded sample into smaller tissue samples. Reduced the ethanol content in FAA so as to reduce the damage to tissue. Three 100% ethanol concentration gradients and extended the dehydration time can fully dehydrated tissues. A little eosin dye was added into the ethanol solution to facilitate the separation of paraffin and sample. Embedding was carried out on an enzyme-free clean bench to avoid damaging to the RNA structure and plant tissue structure.
Preparation of hybridization sections	The commonly used slice thickness is 8 μm.	The tissue section of the sample is too thin.	The slice thickness is 10 μm.	It can increase the number of target molecules in tissue as much as possible
Hybridization pretreatment	The last alcohol concentration gradient is 30%. Several concentration gradients of 0.85% NaCl and ethanol are set.	Multiple reagent solutions are used multiple times at this stage, resulting in a large consumption of resources.	Removed the 30% alcohol concentration gradient and mixed gradient of 0.85% NaCl and ethanol, 0.85% NaCl was directly used.	Reduce the consumption of reagents.
Hybrid incubation	The processing time of Buffer 3 was 30 min.	The result of RNA localization in mature tissue of cucumber seedlings was not accurate.	The processing time of Buffer 3 was extended to 1 h.	Improve the specificity of RNA probe.
Chromogenic reaction microscopy	No emphasis on sticking the slides together.	The specificity of RNA localization in mature tissues of cucumber seedlings was affected.	In the chromogenic reaction, sticking the slides together.	The slides were adhered together to prevent the slices from falling off.
Post-reaction processing	TE buffer was used to stop the chromogenic reaction, and then rinsed with xylene and ethanol according to the gradient, or a picture must be taken after stopping the reaction with clean water.	The hybridization signal is disturbed by a large number of cell contents and cannot be clearly seen, and it is also easily decolorized by rinsing with ethanol or xylene.	The chromogenic reaction was terminated with clean water and then left to dry at room temperature. Finally, each slide was directly mounted with a mixture of 90 μL neutral resin/xylene (1:1).	The samples after mounting were hardly affected by the inclusion impurities, the hybridization signal was clear.

## Abbreviations

AP	alkaline phosphatase
BCIP	5-bromo-4-chloro-3-indole-phosphate
DIG	digoxin
Digoxigenin-11-UTP	biotin digoxin labeled uracil nucleotide
EDTA	ethylene diamine tetraacetic acid
FAA	formaldehyde-acetic acid-ethanol
FAO	Food and Agriculture Organization of the United Nations
GFP	green fluorescent protein
GUS	β-glucuronidase
ISH	in situ hybridization
MS	Murashige and Skoog
NBT	nitroblue tetrazolium chloride
PBS	phosphate-buffered saline
SSC	saline sodium citrate
TBS	tris-buffered saline
NPF	Nitrate/Peptide Transporter Families
